# Broadening horizons: new links between cilia and heart development and disease

**DOI:** 10.3389/fcvm.2026.1699088

**Published:** 2026-02-17

**Authors:** Wenqi Ma, Zhuofeng Zhang, Yun Ma, Chengxu Ma

**Affiliations:** 1 The First Clinical Medical College of Lanzhou University, Lanzhou, Gansu, China; 2 Department of Endocrinology, The First Hospital of Lanzhou University, Lanzhou, Gansu, China

**Keywords:** cilia, cardiovascular disease, congenital heart defects, gene mutation, heart valves, fibrosis, heterotaxy

## Abstract

Congenital heart disease (CHD) is the most common birth defect, and its pathogenesis is closely related to the abnormal establishment of the left-right (LR) bod*y* axis, which highly depends on the ciliary function of the left-right organizer (LRO). This review systematically expounds the molecular pathways by which ciliary structural and functional abnormalities cause cardiac malformations by integrating multi-species model evidence. We believe that defects in multiple conserved genes (including *CFAP45*, *ZIC3*, *FOXJ1*, *NEK3*, *APLNR*, and microRNAs) disrupt ciliary assembly, motility, or signaling capacity, leading to the disappearance of the leftward nodal flow or mechanical sensing failure within the LRO. This further interrupts the left-specific calcium ion flicker and the activation of the Nodal-Pitx2 signaling cascade, ultimately resulting in failed cardiac looping and structural defects (such as ventricular septal defect and transposition of the great arteries). This review integrates transcriptional regulation, protein stability, miRNA-mediated fine regulation, and the planar cell polarity (PCP) pathway into a unified “cilia-LRO-heart” network and explores the molecular mechanisms of cilia in valve diseases and cardiac fibrosis. This not only deepens the understanding of the fundamental biological processes of heart development but also provides new molecular targets and theoretical frameworks for the genetic diagnosis and counseling of related congenital heart diseases.

## 1 Introduction

Congenital Heart Disease (CHD) is the most common birth defect in newborns, with a global incidence rate of approximately 1% (1). Despite significant advancements in surgical and interventional treatments, CHD remains a leading cause of infant mortality, and the genetic and molecular etiology of many cases remains largely unknown (2). In recent years, an increasing body of evidence has indicated that numerous CHD cases result from disruptions in early embryonic development events, with errors in the establishment of the left-right (LR) bod*y* axis being a key and underestimated cause (3).

The establishment of LR asymmetry is a highly conserved and precisely regulated developmental process, at the core of which is a transient organ, the left-right organizer (LRO) (4). In model organisms such as mice, zebrafish, and Xenopus, specialized ciliated cells of the LRO generate a directional extracellular fluid flow (“nodal flow”) and sense this flow, breaking bilateral symmetry and initiating left-specific Nodal-Pitx2 signaling cascades, thereby guiding the asymmetric positioning and morphogenesis of internal organs such as the heart and stomach (5–7). Any genetic defect that disrupts the structural integrity, motility, or signal transduction capacity of LRO cilia can dismantle this initial LR signal, leading to randomization of visceral heterotaxy or isolated cardiac laterality defects (8).

The possible molecular mechanisms mechanism involves a complex multi-level regulatory network. At the structural level, axonemal proteins such as CFAP45 are crucial for maintaining the function of the dynein arms and ciliary motility; their mutations can result in immotile cilia and the disappearance of nodal flow (9). At the transcriptional level, the master regulator NOTO and its downstream targets FOXJ1 are central to initiating the ciliogenesis program, while transcription factors such as *ZIC3* coordinate the correct assembly and positioning of cilia (10–12). Additionally, microtubule deacetylation mediated by kinases (such as the NEK family), nuclear transport maintained by nuclear pore proteins (NUPs), and post-transcriptional fine-tuning by microRNAs (such as *miR-430a*, *miR-103/107*) collectively ensure the normal development and function of cilia (13–16). Recent studies have also revealed that the G protein-coupled receptor *APLNR* and its ligand Apela fine-tune cardiac LR patterning in both cilia-dependent and -independent manners (17).

By reviewing and analyzing the latest genetic, developmental biology, and biochemical evidence, we have constructed a complete pathogenic model from gene mutations to organ malformations. We propose that understanding the specific roles of these genes in the continuous process of “ciliary structure—fluid mechanics—biological signaling—morphogenesis” is of critical importance for revealing the etiology of CHD, improving genetic diagnostic strategies, and developing potential intervention measures.

## 2 Structure and components of Cilia

The cilium is an extracellular organelle located at the cell, covered by a lipid bilayer and attached to the plasma membrane. Cilia can be structurally divided into subcompartments including the basal body, transition zone, axoneme, ciliary membrane and ciliary tip (18). The matrix originates from the mother centriole and consists of protein-based duplex microtubules (19). Upon completion of cell division, the mother centriole is converted into a basal body, triggering ciliogenesis. The mother centriole is modified into a basal body and attached to the plasma membrane by its distal appendage. Once the basal body docks onto the membrane, the nascent cilium becomes a separate compartment separated from the cytoplasmic lysate by a transition zone and, finally, the rest of the axon protrudes from the cell body (18). During this process, the distal appendages of the mother centriole become transition fibres that connect the matrix microtubules to the ciliary membrane. Where the ciliary membrane meets the cell membrane, ciliary pockets are formed and their membrane structure is concave inwards. Unlike other organelles, cilia are assembled only when the cell moves from mitosis into a quiescent and/or differentiated state; conversely, prior to entering the cell cycle the cilia disintegrate (20).

**Figure 1:**
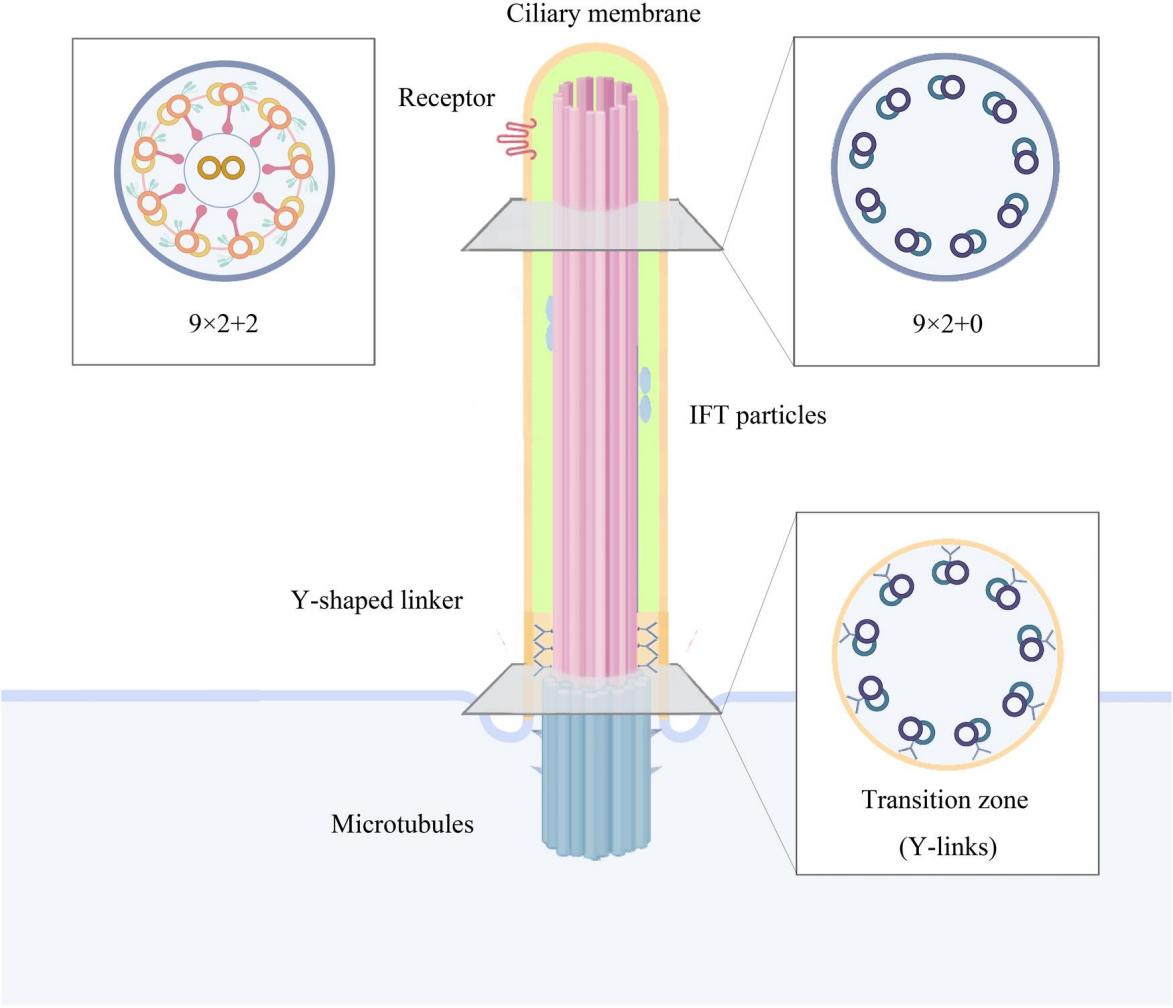
Structure and components of cilia.

The axoneme of the cilium consists of nine sets of duplex microtubules arranged in a cylindrical shape, forming the characteristic “9 + 0/9 + 2” superstructure of the cilium (21, 22). The axon of primary cilia contains a ring of nine outer microtubule bifurcations (known as 9 + 0 axons), whereas the axon of motile cilia has nine outer microtubule bifurcations around two central microtubule monofurcations (known as 9 + 2 axons) and contains associated structures including inner and outer dynamo-protein arms, radial spokes and connexin junctions (23) (Figure 1). Motile cilia with a 9 + 0 microtubule configuration represent a specialised case, residing within a structure historically termed the “embryonic node” or, more recently, the “left–right organiser” (LRO), where they occur as a single cilium per cell. In diverse vertebrate embryos, these solitary LRO cilia play pivotal roles in establishing the embryonic left–right bod*y* axis and subsequently orchestrating the development of fundamental left–right asymmetries in visceral organs such as the heart and gastrointestinal tract (4, 24–26). Axonal bimodal microtubules are continuous with the triadic microtubules of the matrix, and the region of the axon's most proximal connection is known as the transition zone (27). The axoneme and the cilium are connected in the transition zone by Y-connecting fibres. In this region, the transition from matrix to axoneme occurs, and the ciliary membrane appears as a necklace-like structure at the junction under the scanning electron microscope. Together, the Y-connecting fibres and the transition zone form the ciliary gate, which controls the entry and exit of material into and out of the cilium (Figure 1).

In primary cilia, the intraflagellar transport (IFT), Bardet Biedl syndrome(BBsome) and ciliary gate synergistically regulate the entry and exit of substances, suggesting that the primary cilium is a separate chamber connected to the principal cell and composed of specific proteins and lipids (27). Protein transport is carried out internally in the cilium by two endotransporter systems comprising the IFT complex, i.e., retrograde transport by complex IFT-A and paracrine transport by complex IFT-B (28). Proteins are loaded onto IFT granules at the base of cilia in the cytoplasm and translocated through ciliary chamber boundaries in a process known as zonular ciliogenesis (29). The bimodal microtubules of the axon are tracks for IFT, a molecular transport mechanism specific to cilia (30, 31). During IFT, large assemblies of proteins, including cargo proteins and motility complexes, combine to form linear arrays called IFT trains, which traverse along microtubule tracks in two directions (32). The cilium is a large macromolecular machine that is vital for motility, signaling, and sensing in most eukaryotic cells. Its conserved core structure, the axoneme, contains nine microtubule doublets, each comprising a full A-microtubule and an incomplete B-microtubule. Paracrine transport occurs along tubule B and retrograde transport along tubule A (33); this spatial separation prevents collisions between trains travelling in different directions (22). At the same time, IFT is required for the construction and maintenance of cilia as well as for the establishment of cilia-dependent signal transduction pathways (34). A deficiency of the IFT complex leads to shortening or even loss of cilia (35, 36). The BBsome protein complex controls the assembly and cycling of the ciliary matrix and tip IFT complexes, and also manages the entry and exit of a number of proteins into and out of the cilium (37).

### 2.1 The Cilia-mediated signalling pathways

#### 2.1.1 Hedgehog (Hh) signalling pathway

The Hh signalling pathway is a highly conserved that plays a key role in tissue maintenance, renewal and regeneration processes, especially in embryonic development (38). The pathway was first discovered in Drosophila (39), and the protein was named Hedgehog due to its hedgehog-like appearance phenotype in the Drosophila embryo (40). The Hh signalling pathway is mainly composed of Hedgehog ligand, Patched (Ptch) receptor, effector Smoothened (Smo), Gli transcription factor and corrector Suppressor of Fu (Sufu). There are three Hh gene families based on the ligand: Sonic Hedgehog (Shh), Indian Hedgehog (Ihh) and Desert Hedgehog (Dhh). The key site for receiving and transmitting Hedgehog signals is in the primary cilia, where both Smo and Gli proteins localise and function (41).

**Figure 2:**
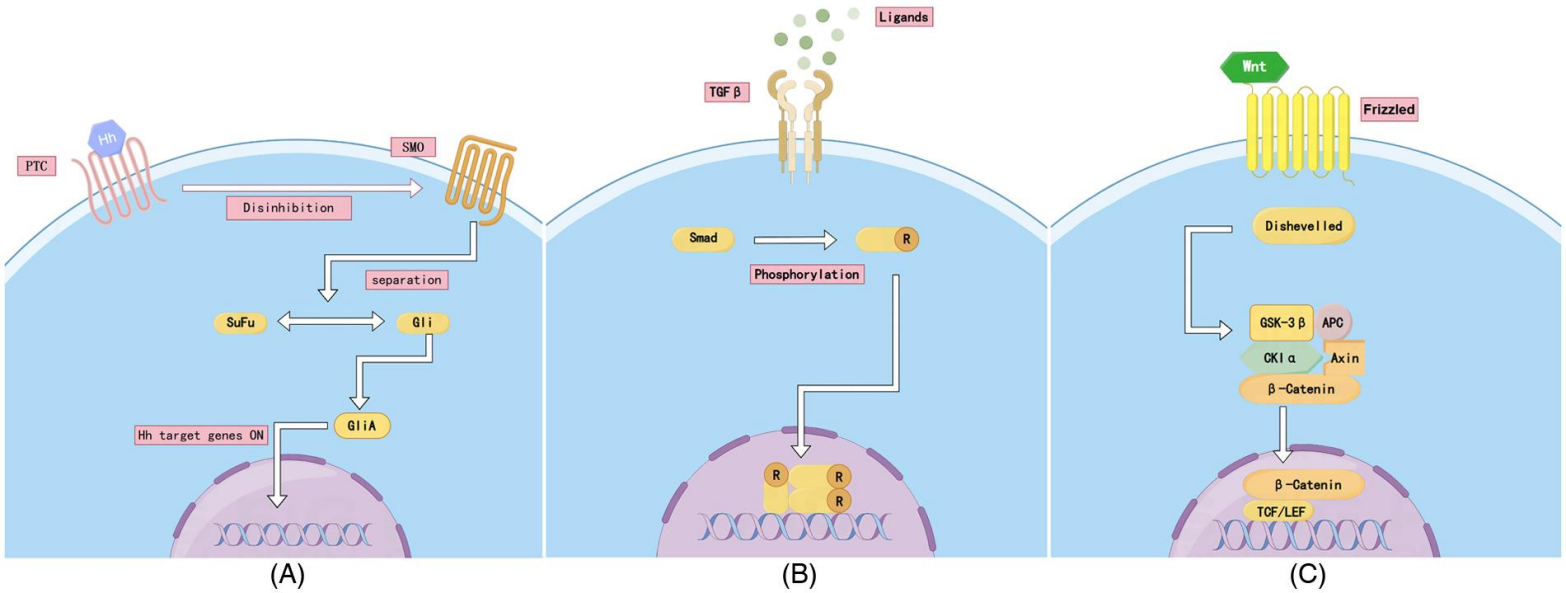
Signalling pathway. **(A)** Hedgehog (Hh) signalling pathway; **(B)** TGF-*β*/BMP signalling pathway; **(C)** Wnt/PCP signalling pathway.

In the absence of Hedgehog ligands (off state), Ptch inhibits Smo and the Gli proteins (Gli1, Gli2 and Gli3) are processed into the inhibitory form of Gli repressor proteins (GliR)—a process normally mediated by protein kinases (e.g., PKA, CK1 and GSK3*β*). The inhibitory form of Gli prevents the transcription of Hedgehog target genes (42) (Figure 2A).

The Hedgehog ligand binds to the Ptch receptor, leading to a conformational change in the receptor, relieving it of its inhibition of Smo and thus triggering intracellular signalling (43–45). Hh signalling is able to be transmitted downstream of Smo by a cytoplasmic protein complex consisting of kinesin (Kif7) and a fusion repressor (Sufu). The activation of Smo leads to the processing of Gli proteins into transcriptional activators GliA (Gli1, Gli2) that induce the expression of Hh target genes and accumulate in the nucleus (46). Finally, GliA migrates to the nucleus and activates the expression of target genes promoting the transcription of genes associated with development, stem cell renewal and tissue patterning (47, 48) (Figure 2A). IFT proteins have been shown to be required for Hh signalling downstream of Ptc1 and upstream of Hh signalling gene targets (49). The deletion of IFT80 prevents Smo localisation to ciliary and inhibits classical Hh signalling (50).

The Hedgehog signalling pathway is essential for the correct patterning of tissues and organs during embryonic development (51). Abnormalities in the Hedgehog signalling pathway can be associated with a wide range of developmental malformations and cancers (52). It also regulates cardiomyocyte differentiation and cardiac tube circulation (53). Silencing CEP104 induces cilia shortening, subsequently disrupting Hedgehog signaling, result in cilia shortening in Kupffer's vesicle of some animals, cardiac laterality and cranial neurodevelopmental defects (54). Membrane ubiquitination pathways regulate Hedgehog signalling and cardiac development, while mutations in genes such as *Megf8* affect Hh signalling (55). This, in turn, leads to cardiac heterotaxy and transposition of the great arteries (TGA) and D- or L-looped heart (56).

#### 2.1.2 TGF-*β*/BMP signal pathway

Members of the transforming growth factor beta (TGF-*β*) superfamily are important regulators of cellular differentiation, phenotype and function and have been implicated in the pathogenesis of many diseases. Their signalling pathways control the development and maintenance of a wide range of tissues. The TGF-*β* signalling pathway can be divided into several different subtypes, mainly including the classical TGF-*β*/Smad pathway and the non-Smad pathway (57).According to the receptor type, the TGF-*β* family includes TGF-*β* (TGF-*β*1, TGF-*β*2 and TGF-*β*3), bone morphogenetic proteins (BMPs), activins, inhibins, anti-mullerian tubular hormones, growth differentiation factors (GDF) and others (58). The BMP signalling pathway is mainly discussed here.

Similar to other signalling pathways, the TGF-*β* signalling pathway consists of TGF-*β* family ligands, receptors and the major signal transduction molecules, Smad family proteins. The BMP signalling pathway, as part of the TGF-*β* superfamily, has a ligand that is a highly conserved secreted glycoprotein (59). The ligand exerts its biological effects by binding to two types of serine/threonine kinase receptors (type I BMPRIs and type II BMPRII), whereas the Smad family proteins (Smad1, Smad5 and Smad8) act as signalling molecules responsible for regulating the expression of specific genes, which affects cellular behaviour (60).

The specific process of the BMP signalling pathway is the selective binding of BMP to BMPRIs and BMPRIIs, leading to the formation of heterodimeric complexes of type I and type II dimers. Similar to other TGF-*β* family members, BMPRIs are substrates for BMPRIIs (61). Therefore, after ligand binding, the type II receptor phosphorylates the GS structural domain, a near-membrane region of the type I receptor rich in glycine and serine residues (62). Upon activation of BMPRI, this receptor initiates intracellular signalling by activating phosphorylated receptor-regulated Smads (R-Smads, i.e., Smad1, Smad5 and Smad8). Phosphorylated R-Smad forms a complex with Smad4 and translocates to the nucleus, where it exerts its effects (63) (Figure 2B).

The TGF-*β* signalling pathway plays an important role in various aetiologies such as coronary artery disease (64, 65), valvular disease (66, 67) and cardiac fibrosis (68, 69). BMPR is a key regulator of normal cardiovascular structure and function (70). Abnormalities in the BMP signalling pathway lead to a variety of cardiovascular abnormalities, with the primary mechanism being the epithelial–mesenchymal transition (EMT). The EMT, and a similar transition that occurs in vascular endothelial cells, is known as endothelial–mesenchymal transition (EndMT). Transcriptional programme switching in EMT is induced by TGF-*β* and signalling pathways mediated by BMP, Wnt-*β*-catenin, Notch, Hedgehog and receptor tyrosine kinases (71). BMP signalling during heart valve development is essential for valve formation, especially heart cushion development, and abnormalities may lead to congenital heart defects and valve disease (72). It has been shown that members of the BMP subfamily exert proinflammatory and anti-inflammatory effects and may regulate fibrosis (73), which plays a partial role in myocardial infarction (74).

#### 2.1.3 Wnt/PCP signalling pathway

The Wnt signalling pathway plays an important role in cardiac development, cell proliferation, differentiation and tissue homeostasis (75), and can be divided into the canonical Wnt/*β*-catenin signalling pathway and the non-canonical Wnt signalling pathway. In the canonical pathway, the Frz receptors and LRP5/6 co-receptors are activated via Wnt ligands; activates Disheveled (Dsh) proteins (76); inhibits the degradation complex consisting of Axin, GSK-3β and APC, which leads to the accumulation of *β*-catenin and its entry into the nucleus; binds to TCF/LEF transcription factors, which activate cell cycle factors including c-Myc and protein D1; and regulates biological processes such as cell proliferation, differentiation and tissue regeneration (77) (Figure 2C).

Non-canonical Wnt pathways include the planar cell polarity (PCP) pathway and the Wnt/Ca^2+^ pathway (78). By the PCP pathway, activated the Dsh protein via the Frz receptor and initiates two signalling branches: one activates the small GTPase Rho, which further activates ROCK via Daam-1, and the other activates Rac and promotes the JNK signalling pathway (79).

The PCP signalling pathway comprises three functional classes: Upstream regulators (e.g., WNT5A/11, SFRPs and WNT inhibitors) establish a tissue-wide WNT activity gradient to induce cellular polarisation, thereby triggering asymmetric localisation of core PCP proteins (though the precise mechanism remains incompletely understood); Core PCP proteins (evolutionarily conserved components including FZD3/6, CELSR1-3, VANGL1/2 and others) orchestrate tissue-level polarity through intercellular interactions; and Downstream effector proteins (cytoskeletal regulators such as RAC1 and DAAM1 activated by DVL1-3), while not asymmetrically localised themselves, exhibit loss-of-function phenotypes identical to core protein deficiencies (e.g., neural tube defects and misoriented stereociliary bundles) and show genetic interactions with upstream regulators (80). Therefore, the comprehensive analysis of these effectors is essential for elucidating the role of PCP signalling in early development (80).

In the Wnt/Ca^2+^ pathway, the heterotrimeric G proteins via the Frz receptor and activates phospholipase C (PLC), which contributes to the release of Ca^2+^ and the activation of PKC, CamKII and calcineurin phosphatase. Elevated levels of Ca^2+^ also activate the NF-AT transcription factor, which regulates gene expression, thereby affecting cell motility and adhesion (81). Dsh proteins also play a role in this pathway, acting as hubs for canonical and non-canonical signalling pathways, enabling the integration and shunting of signal transduction (82).

The Wnt signalling pathway plays a key role in the development, progression and repair of heart disease (83). Its main effects on cardiac function are through the regulation of cell proliferation, differentiation, migration, apoptosis, inflammation and fibrosis (84–87). In myocardial infarction (MI), activation of the Wnt/*β*-catenin pathway promotes inflammatory responses and fibrosis and exacerbates cardiac remodelling in the early stages of myocardial infarction, but moderate modulation promotes cardiomyocyte proliferation and accelerates repair (88). In myocardial fibrosis, the Wnt signalling pathway enhances fibroblast activation and collagen deposition, leading to decreased cardiac compliance, whereas inhibition of this pathway reduces fibrosis and improves cardiac function (89). In arrhythmias, abnormal Wnt signalling pathways may affect the expression of cardiomyocyte gap junction proteins (e.g., Connexin 43), disrupting electrical signalling and increasing the risk of arrhythmogenesis (90, 91). In cardiovascular diseases such as atherosclerosis, hyperactivation of the Wnt pathway promotes vascular smooth muscle cell proliferation, inflammation and vascular calcification, exacerbating atherosclerosis and increasing the risk of cardiovascular events (92, 93). Overall, the role of the Wnt signalling pathway in heart disease is dual and its precise regulation may become a new strategy for cardiovascular disease treatment.

#### 2.1.4 Notch signalling pathway

**Figure 3:**
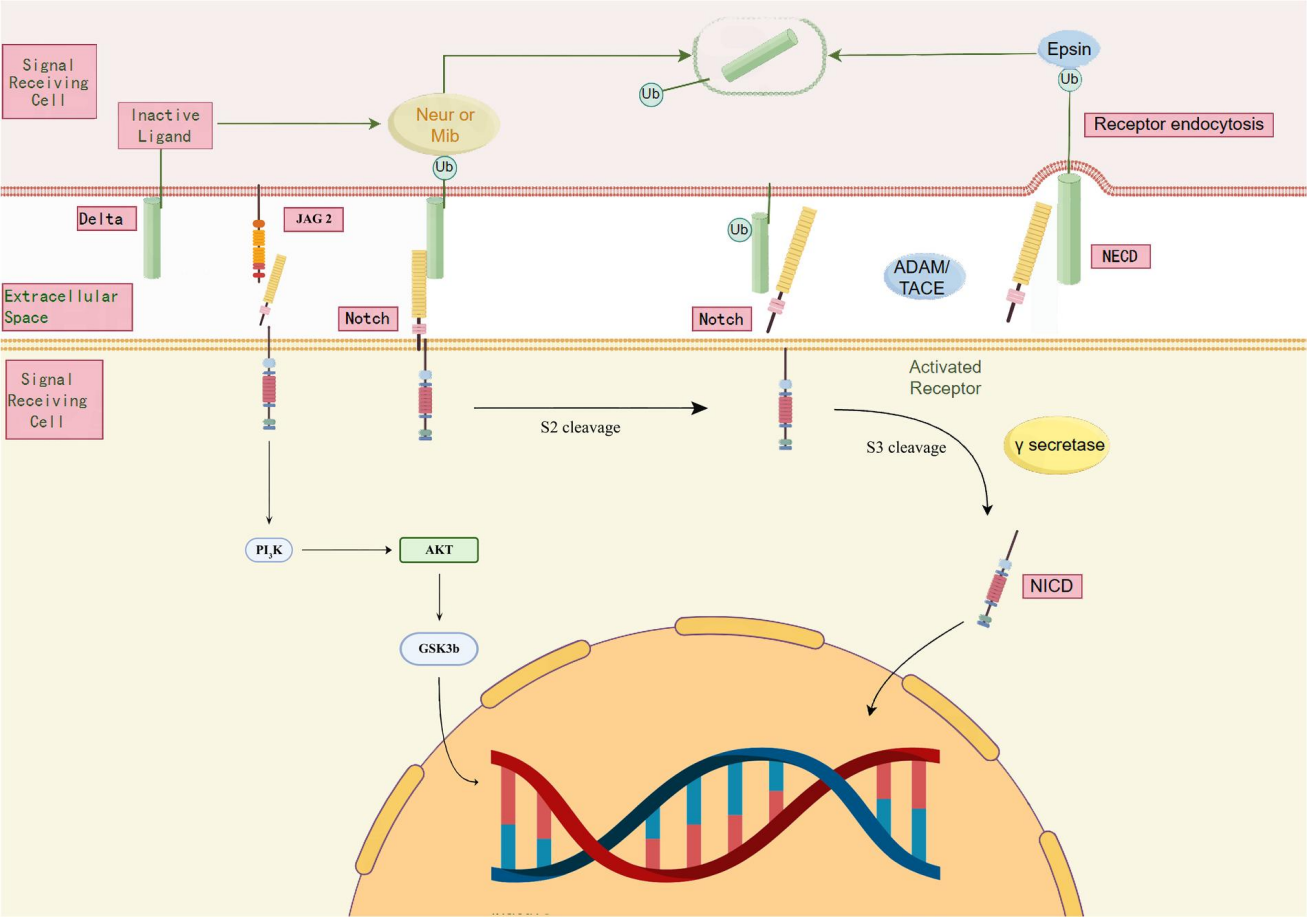
Notch signalling pathway.

Notch signalling is a highly conserved intercellular communication mechanism with an important role in the vascular system and in the development of CHD (94). The Notch receptor family consists of four members, Notch1 to Notch4, which have transmembrane structural domains—with the extracellular portion containing large structures associated with calcium ions (Ca^2+^) and the intracellular portion being shorter (95). Notch receptors initiate signalling by interacting with ligands. During classical signalling, Notch precursors are synthesised and glycosylated in the endoplasmic reticulum, transported to the Golgi apparatus, cleaved at S1 to enter the cell membrane, and finally activated by S2 and S3 cleavage to activate the intracellular structural domains (NICDs), which are translocated to the nucleus to regulate the transcription of target genes (96, 97) (Figure 3).

In the classical model of Notch signalling, the binding of the receptor to a specific ligand triggers endocytosis, leading to a conformational change in the receptor, exposing the S2 site for S3 cleavage and releasing the NICD (98). In addition, Notch signalling is not only carried out via Jagged and Delta family ligands, but may also activate Notch receptors via other molecules (e.g., MAGP1, MAGP2 and YB1), thereby initiating non-classical signal transduction (99). It was also found that the membrane-bolted form of Notch may regulate immune response-associated transcription factors, such as interleukin 10 and interleukin 12, through activation of the PI3K-AKT pathway (100).

The Notch signalling pathway plays a crucial role in cardiac development, regeneration and pathophysiology. It regulates cell differentiation, proliferation and apoptosis and is critical at multiple stages of cardiac development (101). Abnormalities in the Notch signalling pathway are closely associated with a variety of CHDs, particularly during heart valve development (102–104) and ventricular trabeculae (105) formation. Perturbations in Notch signalling may lead to structural defects such as bileaflet aortic valve disease (BAV) and pulmonary stenosis (106). The Notch signalling pathway is essential for regulating cardiomyocyte survival, cardiac fibroblast activation, and cardiac regeneration during MI and cardiac repair (107). It promotes endothelial cell proliferation and neovascularisation and reduces fibrosis (108). It also regulates inflammation and vascular calcification, and prevents the exacerbation of atherosclerotic lesions (109). Targeted modulation of the Notch signalling pathway is therefore considered a potential strategy for cardiovascular disease treatment (110). In cardiac development, Notch signalling is not only a classical transcriptional regulatory pathway, but also involves a number of non-classical regulatory mechanisms.

### 2.2 Ciliary structural and functional abnormalities disrupt developmental signalling pathways

Primary cilia not only serve as signalling hubs but also exert regulatory control over multiple pathways, including Wnt, Hedgehog (Hh) and Notch. Structural or functional defects in cilia can thus profoundly alter downstream gene expression and cellular behaviour. For instance, the discovery of *Inversin*—a key modulator of the planar cell polarity (PCP) branch of non-canonical Wnt signalling—revealed its role in suppressing canonical Wnt/*β*-catenin activity. *Inversin* localises to the ciliary base and its mutations result in ciliopathies such as nephronophthisis (NPHP) and situs inversus (111, 112). Furthermore, the depletion of several cilia-associated genes including *BBS*, *Kif3a*, *Ift88* and *Ofd1* leads to hyperactivation of canonical Wnt signalling, indicating a suppressive role of intact cilia in these pathway (113–115).

Consistently, ablation of primary cilia enhances *β*-catenin signalling even in the absence of Wnt ligands (113, 114), suggesting that cilia act as negative regulators of canonical Wnt signal transduction. Under normal conditions, *β*-catenin is targeted for proteasomal degradation in the absence of Wnt stimuli (116–118), but the deletion of *Kif3a* or *BBS4* results in the accumulation of *β*-catenin regardless of ligand presence (113, 114). Additionally, BBS4 has been shown to interact with proteasome subunits such as the regulatory particle non-ATPase 10, promoting proteasomal localisation near the centrosome (118). These findings support a model in which the primary cilium mediates the proteasome-dependent degradation of *β*-catenin to suppress canonical Wnt signalling (119).

While Wnt signalling is clearly affected by ciliary disruption, the impact on other pathways appears more nuanced. In one study (120), conditional ciliary knockout (cKO) mice exhibited significant reductions in Hh pathway target genes such as *Gli1* and *Ptch1* in neural crest-derived cells (NCCs), while target gene expression in Wnt and Notch pathways—including *Axin2*, *β-catenin*, *Lef1*, *Hey1*, *Hes1* and *Maml1*—remained unchanged (120). This indicates that, among the three pathways, Hedgehog signalling may be most directly and sensitively dependent on ciliary integrity (120).

### 2.3 The role of signalling pathways in cardiac ciliary function

Multiple key signalling pathways regulate cardiac development through mechanisms that involve the primary cilium. The Hedgehog (Hh) pathway is highly dependent on primary cilia, where both Smoothened (Smo) and Gli proteins localise to mediate downstream signalling (41). Intraflagellar transport (IFT) proteins are necessary for transmitting Hh signals downstream of Patched1 (Ptch1). The deletion of IFT80 prevents Smo localisation to the cilium, thereby inhibiting canonical Hh signalling (49, 50). Deficiencies in Hh signalling, such as through CEP104 silencing, cause cilia shortening in Kupffer's vesicles of zebrafish, leading to cardiac laterality defects and cranial neurodevelopmental abnormalities (54). Furthermore, membrane ubiquitination pathways regulate Hh signalling and cardiac development, and mutations in genes such as Megf8 impair Hh signalling, contributing to defects such as TGA and abnormal cardiac looping morphologies (55, 56).

The TGF-*β*/BMP signalling pathway also contributes to cardiac development through mechanisms involving ciliary processes. BMP signalling plays a critical role in heart valve formation, particularly in the development of endocardial cushions, and its dysfunction may lead to congenital heart defects and valve diseases (72). These effects are primarily mediated through the epithelial–mesenchymal transition (EMT) and the related endothelial–mesenchymal transition (EndMT), both of which are regulated by BMP and other signalling pathways that involve ciliary components (71). Additionally, BMP signalling affects fibrosis during myocardial infarction and participates in inflammatory regulation (68, 69, 74).

The Wnt/PCP pathway, part of the non-canonical Wnt signalling network, governs actin cytoskeleton reorganisation and cellular polarity through Rho/ROCK and JNK branches (79). These mechanisms are crucial for the correct localisation of nodal cilia, which in turn determine left–right asymmetry during cardiac development (121).

Although Notch signalling is not exclusively dependent on primary cilia, it plays an essential role in heart valve development, trabecular morphogenesis and cardiomyocyte proliferation—processes that may intersect with cilia-related pathways during cardiac development and disease progression (101–108).

## 3 Cilia play an important role in heart development

The internal organs of vertebrates usually show a marked asymmetry. Under normal circumstances, these organs are arranged in a regular asymmetrical pattern; for example, the heart is usually located on the left side of the body. This regular asymmetry results in a typical arrangement of the internal organs, a phenomenon known as situs solitus (SS). When the normal positioning of organs is not established, lateralised defects such as heterotaxy (Htx) and a completely inverted arrangement of the organs [known as situs inversus totalis (SIT)] occur (122). Htx left–right pattern errors can lead to severe CHD (8).

Vertebrate heart development is closely linked to the formation of the right and left body axes. Embryonic development of the heart requires precise gene expression to coordinate the formation of a four-chambered organ with an asymmetric circulatory system. For example, the human heart consists of four chambers—two atria and two ventricles—which are anatomically asymmetrically connected to the left and right, providing the physiological basis for the exchange of blood in the lung tissues through the pulmonary circulation, as well as for the blood supply to the systemic circulation, and for the conversion of arterial and venous blood (122, 123). Left–right asymmetry is a fundamental feature of biological growth and development. This structural change during development affects the position, pattern and size of multiple organs throughout the body, while the disruption of body symmetry has been a key focus in embryonic developmental research.

### 3.1 Specific role of cilia in cardiac development

Heart formation is a complex developmental process that begins with cardiac progenitor cells expressing the transcription factor *Mesp1*, which originate from the mesoderm in the region of the anterior primitive streak (124). These cells migrate and form bilaterally symmetrical crescent-shaped heart fields in the embryonic midline (125) (the first field is responsible for the formation of the left atrium, right atrium and left ventricle, whereas the second heart field (SHF) is responsible for the formation of the right ventricle and the outflow tract (106), which subsequently converge to form the linear cardiac tube (126). The linear heart tube undergoes a right-handed loop (dextro or D-loop) and develops into a cardiac structure with four chambers. During this process, the endocardial cushion swells and undergoes epithelial-to-mesenchymal transition (EMT) to form heart valves (127), while trabeculae are formed in the heart wall to increase cardiac output and facilitate oxygen exchange (126). In addition, cardiac neural crest cells (128) and epicardial cells (129, 130) are also involved in the formation of cardiac structures and the establishment of coronary circulation, respectively. Eventually, the four-chambered heart is formed at stage E14.5 of mouse embryonic development (131).

Before birth, heart valve development begins with the endothelial–mesenchymal transition (EMT) of the endocardium to form a mesenchymal cushion (132). During late gestation and after birth, these glycosaminoglycan-rich endocardial cushions undergo extracellular matrix (ECM) remodelling (133) and are transformed into stratified leaflets (134). Cell proliferation decreases during heart valve maturation as the ECM is stratified and compacted (135). Prior to valve formation, coordinated contraction of the atria, atrioventricular canal (AVC) and ventricles acts as a primitive valve and drives unidirectional blood flow to form the primitive cardiac tube. However, the shear stress generated by reversed blood flow at the AVC is sensed by endocardial cells and induces the expression of the flow-responsive gene *Krüppel*-like transcription factor 2, which promotes endocardial cushion development (136). Developing heart valves are called endocardial cushions because of their sac-like appearance within the developing heart. These endocardial cushions grow and remodel, eventually forming the valves and membranous septa of the mature heart. The heterogeneity of valve cells and haemodynamics play an important role in this process. Valve development is dependent on the activation of a variety of transcriptional regulators and signalling pathways associated with blood flow, but the mechanotransduction network of the valve requires further study. Primary cilia are present in the endocardial cushion and play a role in coordinating signalling pathways that play an important role in heart valve development, including Notch, SHH, Wnt, PDGF and TGF-*β* signalling (137). Primary cilia in heart valve primordia sense and respond to changes in shear stress to initiate proper valve development (138). Primary cilia are maintained on the surface of endothelial cells at low or turbulent blood flow and are absent in areas of high blood flow such as the endocardial cushion, which may be related to high shear stresses (139). These findings may suggest an association between ciliopathy and valve-related diseases and reveal great research potential in this area.

Primary cilia have been reported to occur throughout embryonic heart development (140). This means that cilia are present in heart cells from the early stages of heart development. In 2008, Slough et al. noted that primary cilia are present in mouse embryonic heart cells at E9.5 (day 9.5 of embryonic development) (139). During heart development, cilia are present on all types of cardiac cells, including cardiomyocytes, endocardial cells, epicardial cells and cardiac cushion cells (6, 139). Cilia on cardiac cushion mesenchymal cells are present in ciliary pockets with different depths and random orientations. The primary cilia of endocardial cells are directed towards the lumen of the inflow and OFT (outflow tract), whereas those of the atria and ventricles are directed towards the blood-filled chambers, and those of the epicardium are directed towards the pericardial chambers (140, 141). However, in 2013 Gerhardt et al. observed a lack of cilia on septal and ventricular cells close to the septum at E10.5 and E12.5 stages of heart development (142). Beyond embryonic development, primary cilia are transiently present in postnatal cardiomyocytes. These cilia persist in embryonic, neonatal and juvenile cardiac tissues but rapidly diminish during cardiac maturation, becoming undetectable in the myocardium of adult rats (143).

### 3.2 The importance of cilia in the body's left–right organisers

The establishment of left–right bod*y* axis patterning is orchestrated by the precisely regulated mechanisms of the left–right organiser (LRO). The key structures determining the left–right bod*y* axis in embryonic development vary across species: the primitive node in mammals, Hensen's node in birds and gastrocoel roof plate (GRP) in Xenopus. In zebrafish embryonic development, this crucial function is mediated by the transient KV, a specialised ciliated organelle (Table 1). KV originates from dorsal forerunner cells (DFCs) during gastrulation (144). These specified cells undergo directed migration along the anterior margin of the embryonic shield before ultimately internalising into the embryo. By the 6-somite stage (6S), KV establishes a fully developed counterclockwise fluid flow system—a dynamic process driven by a cilia-dependent mechanochemical signalling cascade (5, 121, 145, 146).

**Table 1:** Comparative table of Key features in left-right asymmetric development of vertebrates.

Structure/function	Mouse terminology	Human counterparts	Frog terminology	Zebrafish terminology
LRO	Node	Not confirmed (suspected transient structure at the end of the embryo)	Gastrocoel roof plate (GRP)	Kupffer's vesicle (KV)
Ciliary movement pattern	Swivel+Tilt Swing	Unknown (embryo samples not accessible)	Turn clockwise (dorsal view)	Counterclockwise rotation (dorsal view)
Key gene	Pkd2, Dnah5	DNAH5, CCDC39	Dnai1, Dnah9(Zebrafish homologues)	Pkd2, Dnah9, Foxj1a

The LRO exhibits functional heterogeneity in its ciliary system: motile 9 + 0 cilia in the central region generate directional nodal flow, while non-motile 9 + 0 primary cilia in the periphery specialise in mechanosensation (144, 147). This flow induces left-sided Ca^2+^ oscillations through laminar shear stress, establishing a calcium gradient that activates the mechanosensitive channel Pkd2 at cilium tips, triggering left-specific calcium flickers (3, 5, 7). Subsequent Ca^2+^ influx promotes D and 5 mRNA decay to relieve Nodal inhibition (148, 149), ultimately activating the Nodal-Gdf1-Cerl2 and Nodal-Lefty-Pitx2 cascades to drive left-sided gene expression (3, 121, 150–153). Crucially, LRO cellular architecture amplifies this signal: in mice/frogs, motile cilia-bearing cells with small apical surfaces are surrounded by larger cells (154), while zebrafish KV shows dorsoanterior columnar cells (tightly packed) vs. ventroposterior cuboidal cells (large apical surface) (155, 156). This asymmetric organisation enhances LR signalling through ventrally located flow-deflected cilia and dorsally positioned “flow resistor” cilia. Disrupted cellular arrangement causes flow abnormalities and LR defects (156–158). The Wnt/PCP pathway (e.g., Dvl2, Vangl2) mediates posterior basal body migration (5), converting transient flow into stable Nodal-Pitx2 expression—a conserved mechanism whose disruption causes ciliary mispositioning, aberrant flow and laterality defects across species (5–7).

The integrity of this system depends on two critical developmental events: first, KV morphogenesis requires precise regulation of ciliogenesis-related gene expression to ensure proper ciliary density and spatial distribution; second, it depends on maintaining the structural integrity of the embryonic midline (121, 159).

From a biophysical perspective, the core mechanism involves the conversion of mechanical energy (cilia-driven flow) into biochemical signals (Ca^2+^ gradient), which subsequently guide cardiac tube looping through epigenetic reprogramming. This multiscale regulatory mechanism not only ensures spatiotemporal precision for normal dextral looping (D-looping) of the heart tube but also establishes the molecular foundation for subsequent chamber septation and outflow tract positioning (121, 159). Any mutation that causes ciliary structural defects or reduced flow velocity disrupts this mechanochemical coupling, can lead to heterotaxic cardiac development (3, 5).

Studies have shown that over half of CHD-associated genes are linked to ciliary function, including ciliary structural genes, signal transduction genes and genes regulating ciliogenesis (2, 160). Mutations in these genes can lead to defective ciliogenesis or impaired ciliary signalling, resulting in cardiac laterality defects: 90.2% of heterotaxy (HTX) patients exhibit CHD (82.8% with complex malformations), whereas situs inversus totalis (SIT) patients show a lower CHD incidence of 41% (25% with simple malformations). This suggests a positive correlation between the severity of ciliary dysfunction and the complexity of cardiac malformations (161). Research has demonstrated that multiple independently identified CHD-associated genes (e.g., Bicc1, Anks6, Nek8 and Wwtr1) encode proteins that directly interact and form stable complexes. This network-based mechanism provides a molecular foundation for the characteristic complex genetic features of CHD, including incomplete penetrance and phenotypic heterogeneity—whereby distinct heterozygous mutations can collectively disrupt the same functional network to drive disease phenotypes (162). Given the critical role of cilia in cardiac development, it is reasonable to hypothesise that ciliary dysfunction plays a central role in CHD pathogenesis. Any functional or structural defect in the cilia may lead to the formation of cardiac asymmetries, morphological misalignments and valvular abnormalities, all of which are common causes of CHD.

## 4 A New role for cilia in cardiovascular disease

In 2017, Reiter and Leroux proposed classifying ciliopathies into two main groups. The first group of ciliopathies are diseases caused by mutations in cilia-related genes; the second are due to mutations in non-ciliated genes affecting cilia function but not directly involved in cilia structure themselves (163). Of the 303 genes in SCGSv1, 273 were primary cilia genes (90.1%) and 25 were secondary cilia genes (8.3%) (163). The 2021 update of the SYSCILIA Gold Standard (SCGSv2) contains a significant increase of 686 genes over SCGSv1, of which 539 are primary ciliated genes, 133 are secondary ciliated genes and 14 have not yet had their protein localisation reported (and are therefore not designated as either primary or secondary) compared to 383 new gold standard ciliated genes in SCGSv1 (164). In recent years, the molecular level of ciliopathies has been increasingly studied, and multiple pathways have been identified as causative mechanisms.

Ciliogenesis is only one of the many functions that ciliary proteins can perform. Loss of the proteins involved in the processes necessary before and after ciliogenesis will result in the loss of both the previous process and the downstream process (ciliogenesis). Many ciliary proteins have specific locations and functions outside of primary cilia, and ciliary proteins have been found not only in the nucleus and Golgi apparatus, but also in the immune synapse of T cells (165). Extraciliary functions increase the complexity of cilium studies.

### 4.1 Mechanisms of cilia causing CHD

Clinically, patients with CHD often show a higher prevalence of ciliary dysfunction and a large number of *de novo* mutations in their cilia-associated pathways, and in the case of heterotaxy, all known active ciliary mutations are associated with CHD (166).

Cilia Cause Congenital Heart Disease by Disrupting the LR Axis

Djenoune and colleagues demonstrated that cilia are a key structure for sensing node flow by restoring primary cell cilia in Kif3a mutant embryos lacking cilia (3). It has been directly shown that mechanosensing of LRO cilia is both necessary and sufficient for molecular and morphological left–right asymmetry (3). LRO cilia act as mechanically sensitive cellular levers capable of converting biomechanical forces into calcium signals to indicate left–right asymmetry (121, 167). This finding highlights the key role of cilia in cardiac development and the establishment of left–right asymmetry, and that normal cilia function can influence key processes in cardiac development.

#### 4.1.1 CFAP45 (ase CCDC19)

CFAP45 (Cilia And Flagella Associated Protein 45), a key component of the ciliary axoneme, belongs to the family of proteins containing a coiled helix structural domain (168, 169). Biallelic *CFAP45* mutations disrupt ciliary motility and are linked to laterality defects (e.g., heterotaxy) and CHD. In a whole-exome sequencing analysis, researchers identified a pure recessive missense mutation in the *CFAP45* gene in families with CHD, with preexisting diagnoses of heterotaxy syndromes including complex CHD characterised by absence of the spleen, right aortic arch, and unbalanced right dominant atrioventricular canal defects; the patient's older sibling presented with absence of spleen, univentricular pulmonary artery atresia and complete pulmonary venous return abnormality, also consistent with heterotaxy syndrome (8).

CFAP45 deficiency disrupts ciliary function through multiple conserved mechanisms, leading to profound developmental consequences across species. In humans, *CFAP45* mutations cause situs inversus totalis despite a normal axonemal ultrastructure (170). Xenopus laevis models reveal that *cfap45* depletion impairs left–right patterning (evidenced by disrupted pitx2c and dand5 expression) and causes cardiac looping defects with characteristic L-/A-ring abnormalities (8). Structural analyses demonstrate that *cfap45* deficiency leads to axonemal disorganisation (including microtubule malformation and sporadic dynein arm loss), reduced ciliary density/length, and progressive ciliary degeneration manifesting as tip curling, fragmentation and eventual epidermal shedding (8).

In addition, the researchers observed that, where ciliary movement was initially unaffected, the cilia of the African clawed toad became unstable as the cilia matured and beating began. The cilia were first observed to curl at the tip, forming a rounded structure, and then the cilia broke off at different lengths. Eventually, the cilia were shed over the entire surface of the epidermis in a short period of time (8). *CFAP45* deficiency disrupts adenosine diphosphate (ADP) homeostasis and indirectly disrupts the function of the ADP-sensitive regulatory domains of some dynamin ATPases, leading to slower rotational beating in LRO cilia (9). These structural defects correlate with functional impairments, including slowed or undetectable fluid flow. Mechanistically, CFAP45 maintains ciliary beating by regulating AMP-dependent dynamin ATPase activity through adenine nucleotide homeostasis; its deficiency disrupts ADP-sensitive regulation, resulting in slowed rotational beating of LRO cilia (9). This multi-level dysfunction—from molecular perturbation to organ-scale malformation—underscores CFAP45's essential role in ciliary motility and developmental signalling.

#### 4.1.2 NIMA-related kinase family and nuclear pore protein NUP family

The NIMA-related kinase family (Nek1 to Nek11) belongs to the serine/threonine protein kinases, of which Nek1 (171), Nek2 (14) and Nek8 (172) have been shown to be associated with abnormalities of right-to-left symmetry in primary cilia formation and cardiac development. In the pathogenesis of cilia-related CHD, the NIMA-related kinase (NEK) family and nucleoporins (NUPs) play critical roles in cardiac development by regulating ciliogenesis and left–right (LR) asymmetry (173). Biallelic loss-of-function mutations in *NEK3* impair Sirtuin 2 (SIRT2)-mediated *α*-tubulin deacetylation and downregulate the expression of nucleoporins (e.g., NUP205, NUP188, NUP155), thereby compromising ciliary structural integrity and function. This disruption leads to defective LR axis signalling, ultimately contributing to cardiac laterality defects (13, 14). Protein–protein interactions between NUP205 and NEKs may contribute to disease pathogenesis and progression (14, 173).

Current evidence demonstrates that mutations in nucleoporins NUP205 and NUP210 are associated with cardiac left–right patterning defects (14). In zebrafish models, siRNA-mediated knockdown of *nup205* and *nup93* significantly reduced cilia length. Whole-mount immunofluorescence at 48 h post-fertilisation revealed that *nup205* knockdown caused abnormal right–left distribution of cardiac myosin light chain 2 and defective cardiac looping (14). Earlier studies have found that mutations in *NUP20*5 and *NUP210* may be associated with defects in the left–right pattern of the heart (14). The *nup205* and *nup93* siRNA knockdown significantly reduced cilia length, and whole-mount immunofluorescence at 48 h post-fertilisation demonstrated that *nup205* knockdown resulted in significant defects in the right-to-left distribution of cardiac myosin light chain 2 and an abnormal cardiac cycle phenotype (14). Notably, biallelic missense mutations in *Nup205* (p.Thr1044Met and p.Pro1610Arg; NM_015135) exhibited distinct functional consequences: The Pro1610Arg mutant failed to rescue ciliary loss, suggesting a complete loss-of-function allele, while the Thr1044Met mutation, despite impairing protein stability, partially restored ciliary function when overexpressed (13, 174). Further studies confirmed that depletion of *nup93*, *nup188* and *nup205* all resulted in ciliary loss in the left–right organiser (LRO), establishing their essential roles in cilia-dependent establishment of cardiac left–right asymmetry (13). Although NUP62 (175) and NUP98 (176) have been implicated in ciliary function, direct evidence from human diseases or model organisms to establish their definitive association with ciliopathies remains lacking.

#### 4.1.3 *FOXJ1* mutation

*FOXJ1* is a key transcription factor regulating ciliogenesis, and its abnormal function can cause motor ciliopathy (177). In one study, heterozygous variants in *FOXJ1* were identified in a patient with heterotaxy CHD and her mother who exhibited complete sit-to-stand inversion (178). This mutation can cause a motor ciliopathy with features including hydrocephalus, isolated right tachycardia, TGA, chronic obstructive pulmonary disease (COPD) (179) and randomised right and left body asymmetry (11). Clinical exome sequencing revealed a novel truncating *FOXJ1* variant in a patient with complex congenital heart disease (CHD), which was strongly associated with the phenotype (10). Foxj1 is a critical determinant in specifying the cilia used in left–right patterning (180).

*Foxj1*-deficient model organisms exhibit phenotypes associated with primary ciliary dyskinesia and mimic the clinical signs of the patients (phenotypic features of *Foxj1* null embryonic cardiac ring defects include inverted or sinus ring, ventral ring, ventral/sinus ring, ring-less or univentricular heart and anomalous OFTs) (10). *Foxj1*-mutant mice develop premature death with hydrocephalus and left–right body asymmetry randomisation (179). Further studies have confirmed that this variant impairs the ability of *FOXJ1* to promote cilia formation as well as activate downstream genes (10). Regulatory factor X3 (RFX3), a key downstream effector molecule of *FOXJ1*, interacts with *FOXJ1* and acts as a co-activator to regulate the expression of cilia-associated genes. It also plays a guiding role in nodal cilia development and left–right asymmetry specification. *FOXJ1* mutants have a dominant-negative effect on RFX3 transcriptional activity (181). This suggests that RFX3 may be a key intermediate factor in the regulation of cilia formation and functional expression by *Foxj1*. In addition, Noto transcriptional activation of *Foxj1* expression, and therefore its involvement in the regulation of ciliogenesis, is essential for the correct determination of left–right asymmetry, which may explain the abnormal sitting position of some *Foxj1* mutants (11).

We speculate that heterozygous or truncating variants of *FOXJ1* impair its ability to promote ciliogenesis and disrupt the activation of downstream genes (such as RFX3), thereby triggering motile ciliopathy. This leads to the failure of node signalling, resulting in randomised left–right patterning of the heart, viscera and other organs, manifesting as phenotypes such as dextrocardia and TGA.

#### 4.1.4 *Zic3* gene variant

Proteomic analysis of the CPLANE network reveals its role in maintaining ciliary function by regulating ciliogenesis and modulating signal transduction pathways, including intraflagellar transport (IFT), planar cell polarity (PCP) and Hedgehog signalling (182). Defects in associated genes disrupt ciliary trafficking, perturb cellular polarity and cause signalling dysregulation, collectively contributing to ciliopathy-related pathologies (183, 184). *ZIC3*, a member of the zinc finger of the cerebellum (ZIC) family of transcription factors, plays a critical role in left–right (LR) patterning. Mutations in *ZIC3* account for approximately 75% of familial X-linked heterotaxy cases in humans (185), a phenotype recapitulated in *Zic3*-deficient mouse, frog and zebrafish models (186, 187). Although *Zic3* is not expressed in the heart (188), it participates in LR asymmetry establishment through multiple mechanisms: (1) genetically interaction with the Tgf-*β* ligand Nodal (189); (2) modulation of Hedgehog signalling via regulation of GLI3 activity (190). Similarly, *Zic3* may indirectly affect the PCP pathway (through non-classical Wnt cross-regulation), but its genetic interplay in cilia localisation and LR asymmetry has not been clarified (12).

In 2016, Aimee D C Paulussen and colleagues screened 348 patients, including heterotaxy patients and patients with multiple CHDs, for the *ZIC3* gene and identified six variants located in the structural domain of the zinc finger as pathogenic, thus confirming the presence of pathogenic *ZIC3* variants in patients with heterotaxy heart disease (191). *ZIC3*, an X chromosome-encoded zinc finger transcription factor (192), contributes to cardiac development through pathogenic variants (e.g., zinc finger domain mutations) via multiple mechanisms: First, *Zic3* directly participates in embryonic node morphogenesis. Its deficiency leads to structural abnormalities in the node and defective ciliary positioning, thereby disrupting Nodal flow-mediated left–right (LR) signalling (188). Second, nuclear-translocated *Zic3* orchestrates cardiac laterality by functionally integrating with the planar cell polarity (PCP) pathway through multiple mechanisms. Murine studies reveal that *Zic3* modulates PCP signalling via interactions with core component *Vangl2* and downstream effectors RAC1/DAAM1, coordinating directional cardiac looping (12). While demonstrating synergistic activation with RAC1 but not CA-MAPK8 *in vitro*, *Zic3* influences pathway activity through both MAPK8 phosphorylation-dependent and -independent mechanisms. Although direct *Zic3*–*Vangl2* binding remains unverified, current evidence supports transcriptional regulation of PCP components (e.g., Rac1, Daam1) and indirect polarity modulation as plausible mechanisms (12). These findings position *Zic3* and the CPLANE network as critical integrators of cilia-dependent left–right signalling and PCP-driven cellular polarity during cardiogenesis. The multifaceted regulation of LR patterning pathways by *Zic3*—spanning transcriptional control, protein interactions and signalling modulation—presents both challenges and opportunities for elucidating the molecular basis of congenital cardiac defects (12, 193).

#### 4.1.5 Melanoma cell adhesion molecule (MCAM) and c-JunN terminal kinase (JNK)

Melanoma cell adhesion molecule (MCAM) is an independent receptor for fibroblast growth factor 4 (FGF4) and a direct upstream receptor for phospholipase C-*γ* (PLC-*γ*), nuclear factor of activated T-cells (NFAT) and a constitutive activator of JNK. Mechanisms of cilia-related CHD: MCAM and JNK signalling pathways regulate cardiac development through ciliogenesis, left–right (LR) asymmetric patterning and cellular polarity (194). In zebrafish and Xenopus, mcam functions as an fgf4 receptor that mediates vesicular trafficking and activates the JNK/planar cell polarity (PCP) pathway, thereby regulating ciliogenesis and apicobasal polarity-driven lumen formation. Downregulation of mcam leads to shortened cilia and Kupffer's vesicle (KV) dysfunction, resulting in situs inversus or developmental mispatterning of the heart, gallbladder and other organs (194).

JNK, as a member of the mitogen-activated protein kinase (MAPK) superfamily with three *JNK* genes present in vertebrates (*Jnk1*, *Jnk2* and *Jnk3 (*195, 196), is a class of kinases that play important roles in key biological processes such as cellular stress response, apoptosis, differentiation, cyclic regulation, inflammatory response and tumourigenesis (197, 198). JNK, as a downstream effector of PCP signalling, orchestrates left–right (LR) axis establishment through stage-specific regulation by its isoforms (Jnk1/2/3): Jnk1/2 maintain nodal cilia length, promote Nodal flow, and establish the Lefty-1 midline barrier, ensuring proper confinement of left-sided signalling (199). Jnk3 fine-tunes LR patterning by restricting pitx2c expression to the left side, thereby regulating endodermal organ positioning (200).

Loss of JNK function disrupts IFT-B complex stability and the actin cytoskeleton in multiciliated cells, leading to ciliary dysfunction (201). This manifests as cardiac looping defects (e.g., malposition or single ventricle) and great vessel malformations (e.g., transposition) (201). Collectively, these mechanisms demonstrate that the MCAM-JNK pathway critically integrates ciliary dynamics, cellular polarity and LR asymmetric signalling during cardiac morphogenesis. Dysregulation disrupts LR axis specification and organ positioning, ultimately contributing to CHD.

#### 4.1.6 MiR-103/107 and MiR-430a

MicroRNAs (miRNAs) are a class of small non-coding RNA molecules that play important regulatory roles in animals and plants by targeting mRNAs for shear or translational repression (202). MicroRNAs (miRNAs) contribute to cardiac malformations by regulating ciliogenesis, signal transduction and left–right (LR) asymmetric development.

For instance, in zebrafish, *miR-430a* modulates Kupffer's vesicle (KV) development and cardiac laterality by targeting the *sqt* gene (16). Overexpression of *miR-430a* disrupts KV function and cardiac left–right patterning, whereas injection of sqt mRNA rescues this defect (16).

Additionally, the *miR-103/107* family regulates low-density lipoprotein receptor-related protein (LRP) gene expression and targets cilia-associated genes (e.g., *arl6*, *foxH1*, *araf*), coordinating dorsal forerunner cell (DFC) migration, ciliogenesis and downstream nodal signalling (15). Depletion of *miR-103/107* causes developmental abnormalities in both motile and non-motile cilia (e.g., shortened cilia or elongation failure), leading to KV dysfunction and viscero-cardiac misalignment (e.g., cardiac transposition) (15).

Mechanistically, the dysregulation of these miRNAs disrupts cilia-dependent signalling and the spatiotemporal expression of key LR axis regulators, independent of endodermal defects, thereby directly impairing cardiac morphogenesis and driving CHD.

#### 4.1.7 G protein-coupled receptor aplnra/b and ligand apelin

Apelin is thought to be an endogenous ligand for the human G protein-coupled receptor APJ (APLNR), called Apelin receptor (AR/APJ/APLNR) (203). The Apelin/APJ signalling pathway plays a crucial role in cardiac development by regulating ciliogenesis and left–right (LR) asymmetric signalling.

The *aplnra/b* are two genes in zebrafish that are homologues of the human *APLNR* gene. In zebrafish models, the APLNR homologs *aplnra/b* coordinate organ LR patterning in a ligand-dependent manner (e.g., via Apela). Loss of *aplnra* function directly disrupts cilia morphogenesis (e.g., shortened or dysfunctional cilia) in the embryonic Kupffer's vesicle (KV), while also modulating ciliogenesis through upregulation of *foxj1a*, thereby affecting the expression of left-sided genes (e.g., *lefty1*) and cardiac LR positioning (17).

Moreover, APJ receptor signalling (e.g., the *aplnra*-apela cascade) regulates midline signalling in a ciliary-dependent manner during late somitogenesis, whereas *aplnrb* provides compensatory regulation of organ LR patterning in a ciliary-independent manner. Disruption of this pathway leads to KV ciliary dysfunction, causing LR signalling defects and resulting in cardiac malformations (e.g., ventricular septal defects, pulmonary stenosis). Concurrent dysregulation of endodermal differentiation and EMT may further exacerbate structural cardiac anomalies (17).

The central mechanism by which ciliary abnormalities lead to CHD arises from integrated defects in ciliary structure, signalling, and regulatory networks (Table 2). Mutations in *CFAP45*, *NEK3* and nucleoporins (e.g., *NUP205*/*NUP188*) impair ciliary motility (e.g., via disrupted dynein ATPase activity) and microtubule stability, resulting in embryonic nodal cilia rotation failure, disrupted Nodal flow and left–right (LR) signalling breakdown. Pathways such as FOXJ1-RFX3, ZIC3-PCP and *JNK* regulate both ciliogenesis and planar cell polarity (PCP). Their dysfunction causes cardiac looping defects (e.g., transposition of the great arteries, single ventricle). miRNAs (e.g., *miR-430a* targeting *sqt*; *miR-103/107* regulating LRP/cilia-related genes) and the Apelin/APJ receptor system spatiotemporally control LR axis genes (e.g., *PITX2C*), disrupting Kupffer's vesicle (KV) function and cell migration, thereby inducing cardiac misalignment (e.g., ventricular septal defects). Defects in pathways such as MCAM-JNK exacerbate ciliary mislocalisation and cellular polarity dysregulation, collectively impairing cardiac morphogenesis. These multilevel abnormalities stem from decoupled cilia-dependent mechanical signalling (Nodal flow) and chemical signalling (PCP/Hedgehog), ultimately driving complex CHD phenotypes. However, the detailed mechanisms by which these gene variants lead to CHD (congenital heart disease) have not been elucidated yet.

**Table 2:** Regulatory table of cilia-related genes in CHD.

Cilia component	Gene_symbol	Cilia gene? (yes/no/unknown)	Mutant phenotype	Bibliography
Signalling pathway	*SHH*	yes	OFT, AVSD	(212, 213)
	*DHH*	yes	MVP	(167)
	*CEP104*	yes	CHD	(54)
	*Megf8*	no	HTX, TGA, CHD	(56)
Ciliary structure	*CFAP45*	no	CHD, HTX, AVC	(8, 9, 170)
	*Nek3*	yes	HTX	(13, 14)
	*Nup205*	yes	CHD	(14, 174)
Ciliary signalling	*FOXJ1*	yes	CHD, HTX, LRO, OFT	(10, 179)
	*Zic3*	no	VSD, OFT	(12, 188, 191)
	*JNK*	no	CHD	(194, 199)
regulatory networks	*miR-103/107*	no	CHD	(15)
	*miR-430a*	no	CHD	(16)
	*Aplnr*	no	CHD, VSD	(17)
Else	*Dnah11*	yes	HTX, AVSD	(214)
	*Mks1*	yes	AVSD	(214)
	*Ift172*	yes	HTX	(49)
	*EXOC5*	yes	BAV	(218)
	*DZIP1*	yes	MVP	(223)
	*Filamin-A*	no	MVP, cardiac fibrosis	(222)
	*Ift88*	yes	cardiac fibrosis	(240)
	*ALMS1*	yes	cardiac fibrosis	(245)
	*HTRA1*	no	cardiac fibrosis	(246)
	*TULP3*	yes	cardiac fibrosis	(247)

OFT, outflow tract; AVC, atrioventricular canal; CHD, congenital heart disease; HTX, heterotaxy; TGA, transposition of the great arteries; AVSD, atrial septal defect; BAV, bileaflet aortic valve; MVP, mitral valve prolapse; VSD, ventricular septal defect.

Cell adhesion molecule-related/down-regulated by oncogenes (CDON) is a cell surface receptor that is a member of the immunoglobulin superfamily. During embryonic development, CDON regulates the migration of neural crest cells (204). Research has identified CDON expression in dorsal forerunner cells (DFCs) during gastrulation and subsequently in Kupffer's vesicle (KV) epithelial cells during early somitogenesis. CDON deficiency impairs DFC aggregation and reduces DFC population size, ultimately disrupting KV formation, ciliogenesis and organ left–right (LR) patterning (205). In zebrafish models, CDON loss-of-function appears to upregulate Wnt signalling, thereby compromising DFC cohesive migration, KV morphogenesis, cilia formation and proper organ laterality establishment—although the precise molecular mechanisms underlying these phenotypic consequences remain to be fully elucidated (205–207). In addition, numerous studies have identified multiple genes that may be associated with cilia and cardiac development, e.g., *CFAP74 (*208), *DNAH5 (*209), *CCDC141 (*210). However, there is still a lack of sufficient experimental evidence for the specific association of these genes with cilia and cardiac development. Obtaining such evidence may help us to expand our understanding of the cilia's role and function and provide guidance for future directions in cilia research.

### 4.2 Association of cilia with valvular diseas

#### 4.2.1 Atrial ventricular septal defect (AVSD)

Ventricular septal defect (VSD), one of the most common CHDs in humans, is closely associated with disruptions in cilia-dependent signalling networks. The Hedgehog (Hh) signalling pathway exhibits spatiotemporal-specific regulatory functions during mammalian cardiac development: during embryonic stages E7.0–E7.5,the mouse stages, it precisely directs the proliferation of second heart field (SHF) progenitor cells by modulating the Wnt/*β*-catenin pathway and TBX5/FOXF transcriptional network, thereby establishing cardiac left–right asymmetry and influencing ventricular septation (211). The Sonic hedgehog (Shh) signalling subfamily plays a pivotal role in atrioventricular septal morphogenesis, with its deficiency leading to severe malformations such as atrioventricular septal defect (AVSD) and persistent truncus arteriosus (212, 213). Notably, the development of outflow tract (OFT) demonstrates potentially lower dependence on early left–right patterning establishment compared to atrioventricular structures (160).

Genetic studies in mice identified two distinct recessive mutations causing atrioventricular septal defects (AVSD): *Dnah11^avc4^* (affecting motile cilia) and *Mks1^avc6^* (affecting primary cilia structure/signalling). *Dnah11^avc4^* homozygotes developed AVSD only infrequently and strictly in conjunction with heterotaxy (left–right axis defects) (214); it did not disrupt Hedgehog (Hh) signalling in the second heart field (SHF), consistent with the absence of *Dnah11* expression in this tissue (215). In contrast, *Mks1^avc6^* homozygotes consistently developed AVSD independently of heterotaxy and exhibited disrupted Hh signalling within the SHF, where primary cilia signalling genes are highly expressed (214). Whole-exome sequencing (WES) confirmed these as the sole causative homozygous mutations in their respective lines. These findings demonstrate two distinct cilia-dependent pathways to AVSD: (1) an indirect pathway via motile cilia dysfunction causing heterotaxy-associated (syndromic) AVSD (*Dnah11*) (214); (2) a direct pathway via impaired primary cilia-dependent Hh l within the SHF leading to non-syndromic AVSD (*Mks1*) (212). This implicates ciliary genes and Hh signalling components in the genetic basis of both syndromic and non-syndromic human AVSD (214). The mutational spectrum of the intraflagellar transport (IFT) gene *Ift172* further validates this pattern: *Ift172^avc1^* affects only Hh signalling, whereas the *Ift172^wim^* allele causes both signalling defects and visceral heterotaxy (215). Genome-wide SHF transcriptome analysis has confirmed that ciliary motility-related genes are silenced in this region, while structural/signalling genes are highly expressed, thus explaining the decoupling of AVSD and laterality defects in ciliary mutation models. Based on these findings, researchers established a tripartite classification system for ciliary gene mutations: (i) Mutations that affect laterality but not SHF Hh signalling; (ii) mutations that affect SHF Hh signalling but not laterality; and (iii) mutations that affect both laterality and SHF Hh. This model accurately predicts clinical phenotypic combinations (214).

#### 4.2.2 Bileaflet aortic valve (BAV)

BAV is the most common congenital valvular heart defect, with an overall incidence of 0.5%–1.2% (216), and can lead to calcific aortic stenosis. Primary cilia are expressed in a spatiotemporal manner on aortic valve mesenchymal cells, whereas they are rarely observed on the valve endocardium (133). However, recent studies have shown that primary cilia defects are associated with a number of common heart valve defects (137), such as BAV (217) and aortic stenosis (132), which usually become apparent in late adulthood (132). The researchers found a large number of cilia genes from the SysCilia set among the 1,889 SNPs (Single-Nucleotide Polymorphism) most associated with BAV, which accounted for 9.2% of the genes in the set (218). This suggests that ciliary genes may play an important role in the genetics of BAV (6).

Genetic ablation of primary cilia leads to highly penetrant mucinous mitral-aortic valve disease, similar to the cardiac phenotypes observed in ciliopathy patients, and it is hypothesised that cilia may inhibit the differentiation of aortic valve mesenchymal stromal cells (133). These studies identify a morphogenetic link between primary cilia and aortic valve disease and support a model in which primary cilia are not only cellular tentacles but also cellular clocks that determine the timing of activation for differentiation (133). Previous studies have found that EXOC5 (Exocyst Complex Component 5) interacts directly with ARF6 and that ARF proteins (ADP-ribosylation factors) interact with extracellular vesicles to control ciliogenesis and MAPK signalling (134, 135). Recently, a new EXOC5 model has been proposed to study the mechanism of BAV formation and its progression to stenotic valves: deletion of *Exoc5* in mice leads to shorter and fewer cilia within the aortic valve, an increased incidence of BAV, and calcification of the aortic valve, accompanied by an elevation of pERK12, which is a marker of the disease phenotype (218). These findings suggest that primary cilia may play a broad role in the aetiology of BAV. These findings may reveal a potential cilia-dependent pathogenic mechanism that *EXOC5* interacts with ARF6 to regulate ciliogenesis, and its deficiency results in both a shorter ciliary length and a reduced ciliary density, concomitant with MAPK signalling activation—thereby driving bicuspid aortic valve (BAV) pathogenesis.

#### 4.2.3 Mitral valve prolapse (MVP)

Mitral valve prolapse (MVP) affects about 1 in 40 people (219) and can lead to arrhythmias, heart failure and sudden cardiac death, often requiring surgical intervention. At present, there is no clear information about the causes of this disease (220). Genetic studies have similarly shown that congenital heart valve defects may lead to heart valve insufficiency, which precedes mucoviscidosis (MVD) diagnosed later in life (221). An analysis of a large family performed by Katelynn A. Toomer et al. found that the loss of primary cilia during development may lead to progressive mucinous tumour degeneration and severe mitral valve pathology in adults (220), such as BAV (218) and MVP (220).

There is a close correlation between the presence of cilia and the production of specific types of ECM, which play a key role in regulating or responding to changes in the extracellular environment during mitral valve development (222). Genetic ablation experiments confirm the regulatory role of primary cilia on ECM deposition during cardiac development: primary cilia limit ECM production during early valve morphogenesis, and premature loss of cilia leads to dysregulated ECM synthesis. Zinc finger protein 1 (*Dzip1*) mutations lead to changes in ECM synthesis and disruption of homeostasis through cilia loss and increased *β*-catenin signalling, and alterations in ECM homeostasis ultimately lead to the development of a myxomatous phenotype incompatible with normal valve function (220). This new mutation is consistent with the previously identified *DZIP1^S24R^* series of variants, resulting in the reduced stability of DZIP1 and CBY1 and increased *β*-catenin activity (223). Cilium-based signal transduction contributes to the precise regulation of extracellular matrix composition and organisation. Deinhibition of Desert Hedgehog protein (DHH) expressed by endocardial cells at primary cilia, which in turn activates RAC1 kinase via RAC1-GEF, TIAM1, promotes the organisation of *α*-smooth muscle actin and the remodelling of the ECM, which leads to an abnormal enlargement of the valve and the development of a muco-tumourous phenotype, similar to the pathological features of the valve in patients with MVP (167).

Filamin A, a basal body-associated actin-binding protein, partners with MKS3 (Meckelin) to regulate centriole migration and ciliogenesis. It further maintains endothelial integrity and facilitates valvular interstitial maturation post-EMT during valve development (222).

The above-described mechanisms may induce ciliary dysfunction–ECM dyshomeostasis–valvular remodelling, culminating in pathological leaflet enlargement and the development of MVP's hallmark morphological phenotype. Overall, these studies provide new insights into the developmental basis of MVP by revealing alterations in cilia-dependent ECM regulation and suggest that variants in primary cilia genes may be responsible for the disease phenotype in some MVP patients.

### 4.3 Cardiac fibrosis and regenerative remodelling

Cardiac fibrosis is the process of abnormal deposition of non-contractile extracellular matrix in cardiac tissue. Fibrosis is necessary for healing in the acute phase of myocardial infarction, but prolonged cardiac fibrosis leads to cardiac remodelling and impairs cardiac function. Such lesions disrupt the normal structure of the myocardium, interfere with the excitation–contraction coupling of cardiomyocytes, and impair the systolic and diastolic functions of the heart, which may lead to arrhythmias and cardiac dysfunction, and ultimately to heart failure (224, 225).

During cardiac fibrosis, transdifferentiation of fibroblasts into myofibroblasts with secretory and contractile functions is a key cellular event driving the fibrotic response (226). Once epithelial cells and fibroblasts have initiated critical signalling through intact primary cilia, shortening or loss of primary cilia will facilitate the transition and maintain their activity (227). Thus, primary cilia are essential for acquiring and maintaining the myofibroblast phenotype. During EndMT, epithelial and endothelial cells (EC) can transdifferentiate into myofibroblasts (228, 229).

In cardiac fibroblasts, activation of the TGF-*β*1 receptor is a critical step that triggers the production of fibronectin, collagen type I and collagen type III (230). These proteins are important components of the extracellular matrix, and their overproduction and deposition during fibrosis leads to the formation of scar tissue. The transdifferentiation of cardiac fibroblasts to myofibroblasts involves several key steps: initially, activation of TGF-*β* in the cardiac mesenchyme triggers the Smad3 signalling cascade, which promotes the transcription of *α*-SMA (*α*-smooth muscle actin) in the fibroblasts (231). Subsequent changes in the composition and mechanical properties of the extracellular matrix further promote transdifferentiation of myofibroblasts by altering the cellular response to mechanical stress or modulating growth factor signalling (232). Finally, mechanical stress directly stimulates *α*-SMA mRNA synthesis in fibroblasts via the Rho/Rho kinase signalling pathway, but may not be sufficient to independently trigger transdifferentiation of myofibroblasts in the absence of TGF-*β* (233). In the normal heart, fibroblasts are normally protected from mechanical stress by a stable stromal network, and when the structural integrity of this network is compromised, exposure of the cells to mechanical stress may prompt transformation of primitive myofibroblasts (233). Changes in mechanical stress may therefore play a greater role in promoting the transdifferentiation of primitive fibroblasts to myofibroblasts (226, 234). The hyaluronic acid layer surrounding the cells (235), ED-A fibronectin in the region of myocardial infarction (236) and non-fibrillar collagen (deposition of type VI collagen) (232) all contribute to the promotion and maintenance of myofibroblasts in the formation of their characteristic properties (236). Disruption of type VI collagen helps to reduce the fibrosis induced by myocardial infarction, contributes to the recovery of cardiac function (237) and may also lead to a reduction in cardiomyocyte death (237). However, Lefty-1 may prevent fibroblast–myofibroblast transdifferentiation in part by regulating Smad3, JNK-3 and BMP-5 activity in the TGF-*β*/BMP signalling pathway (238).

Ciliated fibroblasts are enriched in areas of myocardial injury and depletion of primary cilia in cardiac fibroblasts reduces collagen production in response to TGF-*β*1 stimulation (239). Studies have shown that the inhibition of intraflagellar transport protein-88 (Ift88) promotes EMT and reduces cardiac remodelling after myocardial infarction. Conversely, knockdown of Ift88 promotes EMT and neovascularisation after myocardial infarction, reduces deleterious remodelling processes and improves cardiac function 3 weeks after myocardial infarction (240). This suggests that the breakdown of primary cilia may affect epicardial EMT and cardiac remodelling in the ischaemic heart. Induction of primary cilia disassembly by Ad-shIft88 treatment in the myocardium promotes epicardial EMT and enhances the expression of Wnt/*β*-linker protein and growth factor (*β*-FGF), thereby improving myocardial neovascularisation, cardiac remodelling and post-infarction function (241).

HTRA1 (High temperature requirement serine peptidase) encodes a serine protease involved in extracellular matrix metabolism and inflammation regulation (242); ALMS1 (Alstrom syndrome 1) encodes a cilia-associated protein that maintains cilia structure and signalling (243); and TULP3 (Tubulin polymerisation promoting protein 3) regulates microtubule and intraciliary transport (244). Abnormalities in all three functions are associated with myocardial fibrosis (245–247). Oxidative stress and endoplasmic reticulum (ER) stress, as direct triggers of myocardial fibrosis, can induce upregulation of *HTRA1* in a variety of diseases (248, 249). Significant attenuation or overexpression of fibrotic protein expression was observed after inhibition or upregulation of *HTRA1* expression in primary cardiac fibroblasts and inability to be activated by transforming growth factor-*β*1 (TGF-*β*1) (250). ALMS1 deficiency leads to distinct ciliary and fibrotic phenotypes through TGF-*β* signalling dysregulation. Following ALMS1 depletion, cells assemble abnormally elongated cilia with increased morphological defects. This is accompanied by downregulation of TGF-*β* signalling, which ALMS1 appears to modulate through both canonical and non-canonical pathways, as evidenced by elevated ALMS1 mRNA levels upon TGF*β*-1 and BMP2 stimulation (251). Fibrosis in ALMS primarily results from ALMS1 mutation-induced defects, with ALMS fibroblasts demonstrating hyperresponsiveness to TGF-*β* stimulation. This leads to excessive extracellular matrix (ECM) deposition due to disrupted ECM homeostasis—where the normal balance between synthesis and degradation is lost in ALMS patients (251, 252). In addition, some studies have found that Alms1 deficiency enhances cell migration of fibroblasts and neonatal cardiac fibroblasts, whereas Alms1 depletion enhances cardiomyocyte proliferative activity (245). The tubby family protein TULP3 has been shown to localise to the plasma membrane, primary cilia and the nucleus (244, 253), and mutations in *TULP3* lead to defective transport of ARL13B (247), INPP5E (254) and GPR161 (255), disruption of cilia composition and, directly or indirectly, to dysregulation of the pro-fibrotic WNT and TGF-*β* signalling pathways (256). Meanwhile, TULP3 interacts directly with Sirtuin 1 (SIRT1), a key regulator of fibrosis (256), and SIRT1 is a well-recognised regulator of TGF-*β*-mediated organ fibrosis (257).

These findings reveal the importance of primary cilia in cardiac remodelling and highlight primary cilia as a potential therapeutic target after myocardial infarction, although the specific cell types responsible for the beneficial effects remain to be further defined (239). They also suggest that pharmacological restoration of primary cilia in atrial fibroblasts may help to inhibit the development of atrial fibrosis and atrial fibrillation (258).

### 4.4 Golgi apparatus (GA) and mitochondria association with cilia

The Golgi apparatus, as a central hub for intracellular cargo sorting and trafficking (259), plays a critical role in maintaining ciliary structure and functional homeostasis. Studies have demonstrated that the Golgi apparatus participates in primary ciliogenesis by regulating membrane protein trafficking (260, 261). For example, *Yif1b* knockout mice exhibit ciliary defects, suggesting that Golgi dysfunction or impaired Golgi–cilia trafficking may underlie these pathologies (262). Cumulatively, these findings highlight that Golgi integrity is essential for normal ciliary function (193, 260, 263).

Mitochondrial dysfunction has emerged as a key contributor to congenital heart disease (CHD). Pathogenic mtDNA variants frequently identified in CHD patient cardiac tissues disrupt mitochondrial biogenesis via interference with pathways such as mTOR signalling, thereby impairing the establishment of embryonic left–right asymmetry (2, 264–266). Mitochondrial defects also cause abnormal length and beating frequency of nodal cilia, perturbing fluid flow in Kupffer's vesicle (KV) and disrupting the spatial expression of key determinants such as DAND5 (218, 267). Thus, precise regulation of mitochondrial activity is required for proper ciliogenesis (265). Mitochondrial genetic mutations expand the CHD pathogenic spectrum by dysregulating cilia-mediated developmental programs (268).

In summary, the Golgi apparatus and mitochondria contribute to cilia-related physiology through distinct mechanisms—membrane trafficking regulation and energy metabolism, respectively. Dysfunction in either organelle disrupts cilia-dependent developmental pathways (e.g., left–right axis determination), ultimately driving CHD pathogenesis. Given the close association between the Golgi, mitochondria and cilia, future ciliopathy research should not only focus on cilia themselves, but should also consider the role played by extraciliary structures in related diseases. At the same time, more attention should be paid to the study of extraciliated proteins.

### 4.5 Prospects for the clinical application of ciliotherapy

The role of cilia in disease is self-evident, so can protocols for targeting cilia to treat related diseases be applied in the clinic? Recent studies have identified the potential of cilia as clinical and preventive therapeutic targets and new nanoparticle technologies that can target primary cilia, control cilia movement and function *in vitro* to improve cardiac function.

Mice deficient in EC-specific IFT88 expression have reduced survival and cardiac function, increased cardiomyocyte vacuolisation, cardiac fibrosis and serum CK-MB levels after adriamycin (DOX (269) treatment (270). The antithyroid drug propylthiouracil (PTU) alters cilia-driven flow and disrupts the normal genetic programme involved in LR axis determination, with PTU exposure associated with abnormal cilia polarisation, abnormal left flow driven by germinal ridge (GRP) cilia, aberrant expression of Coco and Pitx2c (271), and a proportion of cells lacking posteriorly localised monocilia (271) (a decreased percentage of posteriorly localised cilia disrupts blood flow and LR development (272, 273). This suggests that cilia can be used as therapeutic targets.

A new study has designed a iron oxide nanoparticle-based cilia therapy technology to specifically target primary cilia in cultured cells and vascular endothelial cells via CT-Fe2O3-NPs and enable remote control of cilia movement and function via an external magnetic field (274). This nanoparticle technology significantly improved cardiac function in a model of ciliopathic hypertension, including lowering blood pressure and improving left ventricular pressure, output per beat, ejection fraction and total cardiac output (274). In addition, resveratrol activates SIRT1, which binds to Smad2/3 and inhibits the nuclear translocation of Smad2/3, which in turn regulates EndMT *in vivo* and *in vitro* via the TGF-*β*/Smad2/3 pathway, and can attenuate isoproterenol-induced cardiac fibrosis (257).

These studies show that cilia therapy offers the possibility of personalised medicine for patients with ciliopathies, maximising therapeutic benefits and reducing side effects through specialised drug delivery.

## 5 Outlook

Technological advances have revealed the critical role of cilia in cardiac development and function, particularly in shaping cardiac asymmetry. However, we still know little about the specific roles of cilia in cardiac development, and further research is needed to elucidate their complex functions in cardiac health and disease. Of particular note, the role of cilia in CHD may affect a wider range of organs and tissues, implying that they play a more critical role in human health than is currently recognised. In addition, cilia–gene interactions and mitochondrial connections provide opportunities to explore new treatments for ciliopathies. Activation or inhibition of cilia may provide new directions for the treatment of congenital and acquired heart diseases. As interdisciplinary research advances, we may witness everything from the complexity of gene–protein interactions to in-depth explorations of CHD–cilia associations, as well as the development of personalised medicine and early diagnostic techniques. These studies will drive innovation in personalised treatments and ultimately improve the prognosis for patients with cilia-related diseases.

## 6 Conclusions

Overall, we summarised the latest findings to make the case for the pathogenic potential of cilia in parafascial defects and ciliopathies, with the aim of providing further insight into the mechanisms of ciliopathology in patients with human CHD and ciliopathies, expanding understanding of the complex genetics of ciliopathies, and helping to elucidate the underlying mechanisms of various clinical features.
